# Differential Expression of Osteo-Modulatory Molecules in Periodontal Ligament Stem Cells in Response to Modified Titanium Surfaces

**DOI:** 10.1155/2014/452175

**Published:** 2014-06-25

**Authors:** So Yeon Kim, Ji-Yeon Yoo, Joo-Young Ohe, Jung-Woo Lee, Ji-Hoi Moon, Yong-Dae Kwon, Jung Sun Heo

**Affiliations:** ^1^Department of Maxillofacial Biomedical Engineering and Institute of Oral Biology, School of Dentistry, Kyung Hee University, 26 Kyunghee-daero, Dongdaemun-gu, Seoul 130-701, Republic of Korea; ^2^Department of Oral & Maxillofacial Surgery, School of Dentistry, Kyung Hee University, 26 Kyunghee-daero, Dongdaemun-gu, Seoul 130-701, Republic of Korea

## Abstract

This study assessed differential gene expression of signaling molecules involved in osteogenic differentiation of periodontal ligament stem cells (PDLSCs) subjected to different titanium (Ti) surface types. PDLSCs were cultured on tissue culture polystyrene (TCPS), and four types of Ti discs (PT, SLA, hydrophilic PT (pmodPT), and hydrophilic SLA (modSLA)) with no osteoinductive factor and then osteogenic activity, including alkaline phosphatase (ALP) activity, mRNA expression of runt-related gene 2, osterix, FOSB, FRA1, and protein levels of osteopontin and collagen type IA, were examined. The highest osteogenic activity appeared in PDLSCs cultured on SLA, compared with the TCPS and other Ti surfaces. The role of surface properties in affecting signaling molecules to modulate PDLSC behavior was determined by examining the regulation of Wnt pathways. mRNA expression of the canonical Wnt signaling molecules, Wnt3a and *β*-catenin, was higher on SLA and modSLA than on smooth surfaces, but gene expression of the calcium-dependent Wnt signaling molecules Wnt5a, calmodulin, and NFATc1 was increased significantly on PT and pmodPT. Moreover, integrin *α*2/*β*1, sonic hedgehog, and Notch signaling molecules were affected differently by each surface modification. In conclusion, surface roughness and hydrophilicity can affect differential Wnt pathways and signaling molecules, targeting the osteogenic differentiation of PDLSCs.

## 1. Introduction

Titanium (Ti) substrates are commonly used as biomaterials in dental implantology because they provide excellent biocompatibility for peri-implant bone formation. Many clinical and experimental studies have demonstrated that surface properties, such as topography, roughness, surface energy, and hydrophilicity, are pivotal factors in enhancing osseointegration [1, 2]. Although surface roughness and hydrophilicity remain the major variables determining cell response, different types of cell derived from various tissues also react differently to surface properties [3, 4].

Preliminary assessments of potential biomaterials are often made using osteoblasts, osteoblast-like cells, or bone marrow-derived mesenchymal stem cells [5, 6]. Periodontal ligament stem cells (PDLSCs) are attractive for assessing osseointegration between titanium implants and bone tissue because they are known to self-renew, differentiate into multiple lineages, and function in periodontal tissue regeneration [7]. Moreover, PDLSCs can be obtained more readily than other adult stem/progenitor cells (e.g., bone marrow-derived mesenchymal stem cells or osteoblasts, which are commonly used in implantology).

Studies using various cell culture models have shown different biological behaviors of cells reflecting differences in surface properties [8, 9]. In a recent study, the cell spreading, survival, and* in vitro* osteogenic differentiation of an immortalized human PDL cell line cultivated on two Ti scaffolds with different topographies were analyzed; the responses of these cells differed from those of osteoblasts, suggesting the cell-type specificity of responses to different surface structures [10]. However, the mechanism of the physiological transition between the nonphysiological Ti surface and surrounding cells has not been determined. Moreover, considering the biological role of PDLSCs in osteogenic differentiation, the characterization of their responses to Ti surfaces with different topographies and hydrophilicities is important.

Thus, in the present study, we first tested Ti substrates using PDLSCs to demonstrate the usefulness of this model for novel strategies in PDL engineering and secondly classified the influence of different topographies and hydrophilicities of Ti surfaces on the expression of various functional factors in PDLSCs involved in osteogenesis in the absence of osteogenic supplements, and finally we evaluated biomarkers of cellular activity, including the expression of transcription factors and signaling molecules of PDLSCs on the Ti surfaces.

## 2. Materials and Methods

### 2.1. Materials

Fetal bovine serum (FBS) was purchased from Gibco-BRL (Gaithersburg, MD, USA). Collagen type I (COLIA), osteopontin (OPN), *β*-actin, goat anti-mouse, and goat anti-rabbit antibodies were supplied by Santa Cruz Biotechnology (Santa Cruz, CA, USA). Unless otherwise specified, chemicals and laboratory wares were from Sigma Chemical Company (St. Louis, MO, USA) and Falcon Labware (Becton-Dickinson, Franklin Lakes, NJ, USA), respectively.

### 2.2. Surface Characterization of Titanium

Ti discs with 15 mm diameters and 1 mm thicknesses to fit a 24-well tissue culture plate were prepared and supplied by Institut Straumann AG (Basel, Switzerland). The water contact angle was determined tensiometrically with a telescopic goniometer (Phoenix 300; SEO, South Korea). The morphologies of the PDLSCs growing on Ti discs were examined by scanning electron microscopy (SEM; S-2300; Hitachi, Japan). The discs were washed with phosphate-buffered saline (PBS) and fixed with 2.5% glutaraldehyde in 0.1 M phosphate buffer (pH 7.3) for 30 min and 1% osmium tetroxide in 0.1 M cacodylate buffer (pH 7.4) for 1 h. The discs were then washed with PBS three times, dehydrated through a graded ethanol series, placed in a 100% ethanol bath, and rinsed three times. They were dried and sputter coated with gold (Eiko IB, Japan) and then observed by SEM. Photographs were taken at 15 kV using various magnifications and angles. The surfaces of the Ti were also analyzed using an atomic force microscope (AFM) (XE-100; PSIA Inc., Suwon, Korea) in noncontact mode. The AFM observation was measured at an ambient temperature under a 0.5 Hz scan rate. Digital NC-AFM images were acquired by using XEI 4.1.1 program. Seven measurements were performed at PT and SLA implant; they included height-descriptive parameters, Sq: root mean square roughness, Ssk: skewness, Sku: kurtosis, Sp: maximum peak height, and hybrid-descriptive parameters, Sdq: root mean square surface slope, Sdr: developed interfacial area ratio.

### 2.3. Periodontal Ligament Stem Cell Culture

Periodontal ligaments were obtained from extracted human molars donated by the Department of Oral and Maxillofacial Surgery, Kyung Hee University. All subjects involved in this study were informed about its purpose and procedures, and the study was approved by the Review Board of Kyung Hee University. Written informed consent was obtained from all donors and guardians on behalf of minor participants.

Periodontal ligaments were collected from the middle thirds of roots and cultured in *α* minimal essential medium (*α*-MEM; Invitrogen, Carlsbad, CA, USA) containing 10% FBS, penicillin (100 U/mL), and streptomycin (100 *μ*g/mL; Sigma Chemical Company) according to a previously described method [11, 12]. After two passages, the cells were subjected to magnetic isolation with antibodies to detect the STRO-1 antigen (mesenchymal stem cell marker; Millipore, Billerica, MA, USA) and magnetic beads (Miltenyi Biotec, Germany). The resulting STRO-1(+) cell population was cultured in *α*-MEM plus 10% FBS at 37°C with a humidified gas mixture of 5% CO_2_/95% air. All experiments were carried out with passage 4–7 cells.

### 2.4. Alkaline Phosphatase Activity

ALP activity was performed as previously described [12]. Briefly, Cells were washed twice with PBS and lysed in 50 mM Tris-HCl buffer (pH 7.0) containing 1% (v/v) Triton X-100 and 1 mM phenylmethylsulfonyl fluoride. Total protein was then quantified using the Bradford procedure [13]. The entire cell lysate was assayed by adding 200 *μ*L* p*-nitrophenylphosphate (Sigma Chemical Company) as a substrate for 30 min at 37°C. The reaction was stopped by adding 3 M NaOH and the absorbance was read spectrophotometrically at 405 nm. The enzyme activity was expressed as mM/100 *μ*g protein.

### 2.5. RNA Isolation and Real-Time Reverse-Transcriptase Polymerase Chain Reaction

This process was performed as described in our previous study [12]. Total RNA was extracted from the cells using TRIzol reagent (Invitrogen), following the manufacturer's protocol. Real-time quantification of RNA targets was then performed with a Rotor-Gene 2000 real-time thermal cycling system (Corbett Research, Australia) using a QuantiTect SYBR Green reverse-transcriptase polymerase chain reaction (RT-PCR) kit (Qiagen, CA, USA). The reaction mix (20 *μ*L) contained 200 ng total RNA, 0.5 *μ*M of each primer, and appropriate amounts of enzymes and fluorescent dyes, as recommended by the supplier. The Rotor-Gene 2000 cycler was programmed as follows: 30 min at 50°C for reverse transcription, 15 min at 95°C for DNA polymerase activation, 15 s at 95°C for denaturing, and 45 cycles of 15 s at 94°C, 30 s at 55°C, and 30 s at 72°C. Data were collected during the extension step (30 s at 72°C). The PCR reaction was followed by melting curve analysis to verify the specificity and identity of the RT-PCR products; this analysis can distinguish specific PCR products from nonspecific PCR products resulting from primer dimer formation. The temperature of the PCR products was increased from 65°C to 99°C at a rate of 1°C/5 s, and the resulting data were analyzed using the software provided by the manufacturer. The primer sequences are listed in Table 1.

### 2.6. Western Blot Analysis

Western blot analysis was conducted as previously reported [12]. Protein extract samples (20 *μ*g) were separated by 8–10% sodium dodecyl sulfate polyacrylamide gel electrophoresis and blotted onto polyvinylidene difluoride membranes. The blots were washed with TBST [10 mM Tris-HCl (pH 7.6), 150 mM NaCl, 0.05% Tween-20], blocked with 5% skim milk for 1 h, and incubated with the appropriate primary antibodies (anti-COLIA, anti-OPN, or anti-*β*-actin; Santa Cruz Biotechnology) at the dilutions recommended by the supplier. The membranes were then washed and the primary antibodies were detected with goat anti-rabbit immunoglobulin G (IgG) or goat anti-mouse IgG conjugated to horseradish peroxidase. The blots were developed with enhanced chemiluminescence (Santa Cruz Biotechnology) and exposed to X-ray film (Eastman-Kodak, Rochester, NY, USA).

### 2.7. Immunofluorescence Staining

Cells were fixed and treated with mouse anti-COLIA or anti-OPN antibody (1 : 100; Santa Cruz Biotechnology) for 1 h at room temperature. Fluorescein isothiocyanate-conjugated anti-mouse IgG (1 : 100) was then added for 1 h at room temperature. as previously reported [12]. Images were obtained using a fluorescence microscope (Fluoview 300; Olympus).

### 2.8. siRNA Transfection

Cells were transfected for 24 h with a Stealth small interfering RNA (siRNA) specific to *β*-catenin (5′-CCC UCA GAU GGU GUC UGC CAU UGU A-3′, 200 pmol/L; Invitrogen) or an unrelated control siRNA targeting the green fluorescent protein (5′-CCA CTA CCT GAG CAC CCA GTT-3′), using the Lipofectamine 2000 according to the manufacturer's instructions. as previously described [12].

### 2.9. Statistical Analysis

All data are expressed as means ± standard deviations. One-way analysis of variance was used for multiple comparisons (Duncan's multiple range test). Analyses were performed with the SPSS software (ver. 10.0; SPSS Inc., Chicago, IL, USA). A *P* value <0.05 was considered to indicate statistical significance.

## 3. Results

### 3.1. Surface Characteristics

**Figure 1:**
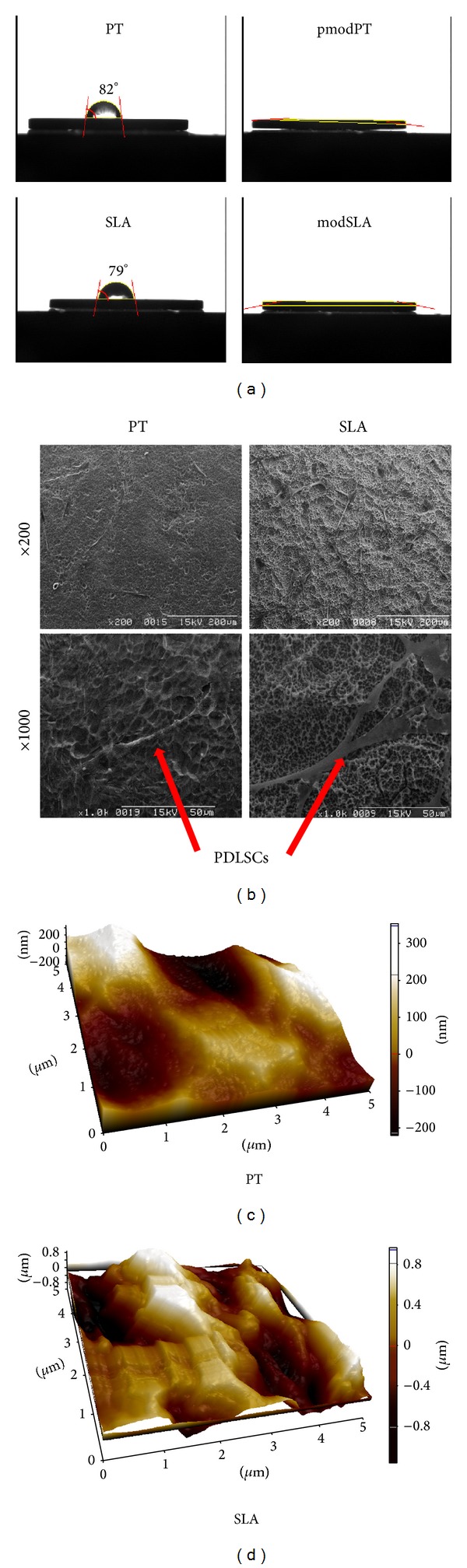
Characterization of titanium substrates. (a) The water contact angles of pretreatment (PT), hydrophilic PT (pmodPT), sand-blasted, large-grit acid-etched (SLA), and hydrophilic SLA (modSLA) substrates were assessed. (b) Topographical features of PT and SLA substrates were examined by scanning electron microscopy at ×200 (upper panels; scale bar = 200 *μ*m) or ×1000 (lower panels; scale bar = 50 *μ*m) magnification. The AFM images of (c) PT and (d) SLA.

The PT and SLA surfaces showed water contact angles of 82.23° and 79.22°, respectively, whereas the contact angles of pmodPT and modSLA surfaces were close to 0°, indicating that the PT and SLA surfaces were hydrophobic, while the pmodPT and modSLA substrates were hydrophilic (Figure 1(a)). SEM images showed morphological differences between the PT and SLA surfaces; the PT surfaces were smooth and planar in comparison with the SLA substrates, consistent with previous reports (Figure 1(b)). The surface roughness of PT and SLA was evaluated by AFM (Figures 1(c) and 1(d)). As shown in Table 2, profile topography measurements revealed significant differences of roughness between PT and SLA implants.

### 3.2. Effect of Surface-Modified Ti Implants on Osteogenic Differentiation of PDLSCs

**Figure 2:**
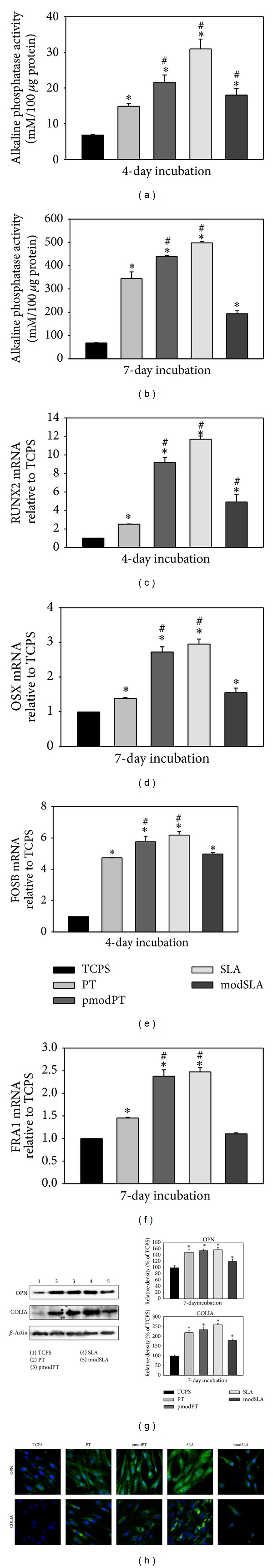
Osteogenic activity of periodontal ligament stem cells in response to titanium surfaces. Cells were cultured on pretreatment (PT), hydrophilic PT (pmodPT), sand-blasted, large-grit acid-etched (SLA), or hydrophilic SLA (modSLA) substrates for 4 or 7 days, and ALP activity ((a), (b)), real time RT-PCR ((c)–(f)), Western blot (g), and immunofluorescence staining (h) of osteogenic markers were then assessed as described in Section 2. Reported values are the means ± standard deviations of five independent experiments. Panels (bars) denote the means ± standard deviations of five experiments for each condition, determined from densitometry relative to *β*-actin. *P < 0.05 versus control (tissue culture polystyrene); ^#^ *P* < 0.05 versus PT substrate. Nuclei were stained with DAPI (blue). A representative result from three independent experiments is shown.

To confirm the effects of surface topography on the biological responses of PDLSCs, the cells were cultured on tissue culture polystyrene (TCPS), PT, pmodPT, SLA, and modSLA surfaces, and alkaline phosphatase (ALP) activity was then assessed 4 and 7 days after induction to identify surface-specific osteogenic differentiation of PDLSCs. ALP activity was significantly higher in cells cultured on all Ti surfaces compared with the control TCPS (Figures 2(a) and 2(b)). In particular, the highest ALP activity appeared in cells on the SLA surface. Interestingly, more ALP activity was observed on hydrophilic pmodPT than on hydrophobic PT surfaces, whereas more activity was observed on hydrophobic SLA than on hydrophilic modSLA surfaces.

To further support the effect of surface properties on PDLSC behavior, we determined the mRNA expression of known osteogenic target genes (runt-related gene 2, osterix, FOSB, and FRA1) using real-time RT-PCR. According to ALP activity, mRNA expression of each osteogenic factor was increased on all Ti surfaces compared with the control TCPS. The expression of all genes was highest on SLA surfaces (Figures 2(c)–2(f)). We also analyzed the Ti surface effect on the osteogenic differentiation of PDLSCs by following the protein level data of osteogenic markers (OPN and COLIA) on day 4 of osteogenic induction. Western blot analysis showed that the level of each protein was increased in cells cultured on all Ti surfaces compared with TCPS. The pattern of protein expression levels in response to each surface was consistent with data from real-time RT-PCR (Figure 2(g)). Moreover, immunofluorescence staining for OPN and COLIA confirmed that PDLSCs on Ti surfaces showed enhanced differentiation into the osteogenic lineage (Figure 2(h)).

### 3.3. Effect of Ti Roughness and Hydrophilicity on Gene Expression of Signaling Molecules

**Figure 3:**
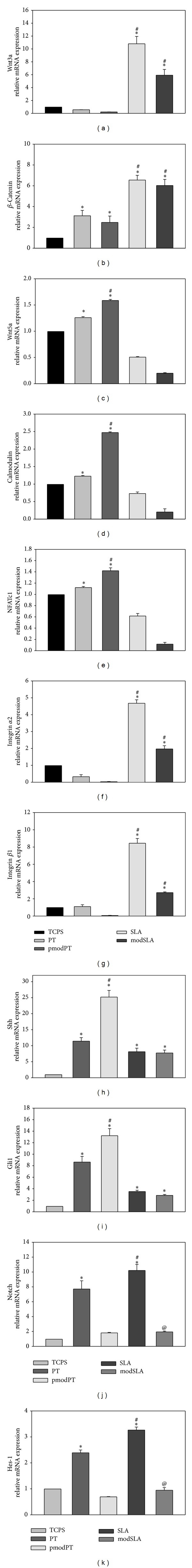
Effect of titanium surface structure on Wnt, integrins, Shh, and Notch signaling molecules. Cells were cultured on each substrate and the mRNA levels of (s) Wnt3a, (b) *β*-catenin, (c) Wnt5a, (d) calmodulin, (e) NFATc1, (f) integrin *α*2, (g) integrin *β*1, (h) Shh, (i) Gli1, (j) Notch, and (k) Hes-1 were analyzed using real-time RT-PCR after 4 days of culture. The values reported are the means ± standard deviations of five independent experiments. *P < 0.05 versus control (tissue culture polystyrene); ^#^ *P* < 0.05 versus pretreatment substrate; ^@^ *P* < 0.05 versus sand-blasted, large-grit acid-etched substrate.

In comparative experiments to determine the role of surface property in the variation in possible signaling molecules during osteogenic differentiation of PDLSCs, we first found that mRNA expression levels of the canonical Wnt signaling molecules, Wnt3a and *β*-catenin, were higher on SLA and modSLA surfaces than on TCPS and smooth surfaces. In contrast, gene expression of the calcium-dependent Wnt signaling molecules Wnt5a, calmodulin, and NFATc1 was increased significantly on PT and pmodPT surfaces compared with TCPS, but it was downregulated on SLA and modSLA surfaces in comparison with TCPS and PT surfaces (Figures 3(a)–3(e)). The mRNA expression of the adhesion molecules integrin *α*2 and *β*1 increased with surface roughness in PDLSCs (Figures 3(f) and 3(g)). Moreover, sonic hedgehog (Shh) expression was slightly increased on SLA and modSLA surfaces but much more increased on PT and pmodPT surfaces (11-fold and 25-fold versus TCPS; *P* < 0.05). Gene expression of the transcription factor for Shh, Gli1, was also increased significantly on smooth substrates (8-fold for PT and 13-fold for pmodPT versus TCPS; *P* < 0.05), but it was decreased on SLA and modSLA surfaces in comparison with PT (Figures 3(h) and 3(i)). However, mRNA expression of Notch and its target gene Hes-1 was increased markedly in PDLSCs cultured on hydrophobic PT and SLA surfaces (7.7-fold and 10-fold versus TCPS for Notch; 2.3-fold and 3.3-fold versus TCPS for Hes-1; *P* < 0.05), but it was unchanged on hydrophilic PT and SLA surfaces compared with TCPS (Figures 3(j) and 3(k)).

### 3.4. Relationships among Wnt Signaling, Integrin, Shh, and Notch during PDLSC Osteogenesis

**Figure 4:**
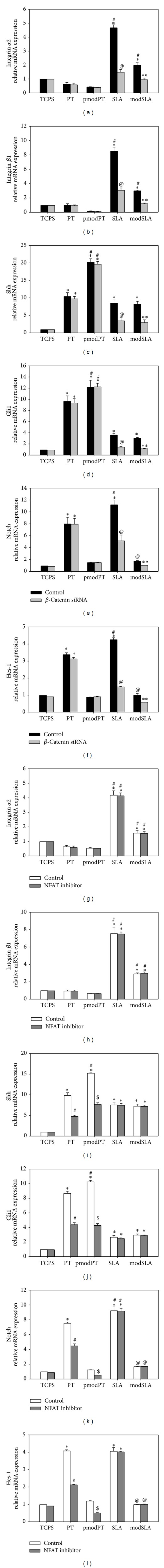
Effect of *β*-catenin knockdown or NFAT inhibitor on integrins, Shh/Gli, and Notch/Hes-1 gene expression. mRNA expression levels of integrin *α*2, integrin *β*1, Shh, Gli1, Notch, and Hes-1 were analyzed after cells were transfected with *β*-catenin-specific siRNA for 48 h or treated with NFAT inhibitor VIVIT (500 nM). A representative result from four independent experiments is shown. *P < 0.05 versus control (tissue culture polystyrene); ^#^ *P* < 0.05 versus pretreatment substrate; ^$^ *P* < 0.05 versus modified PT substrate; ^@^ *P* < 0.05 versus sand-blasted, large-grit acid-etched (SLA) substrate;**P < 0.05 versus modified SLA substrate.

To determine whether the changes in integrins, Shh, and Notch expression were dependent on canonical Wnt signaling, cells were transfected with *β*-catenin siRNA. Knockdown of *β*-catenin by siRNA transfection blocked the increases in integrin *α*2, integrin *β*1, Shh, Gli1, Notch, and Hes-1 gene expression of cells on SLA and modSLA surfaces but did not affect those genes on PT or pmodPT surfaces (Figures 4(a)–4(f)). On the other hand, treatment of NFAT inhibitor diminished Shh, Gli1, Notch, and Hes-1 gene expression of cells on PT and pmodPT surfaces, but there were no changes of each gene on SLA and modSLA (Figures 4(g)–4(l)).

**Figure 5:**
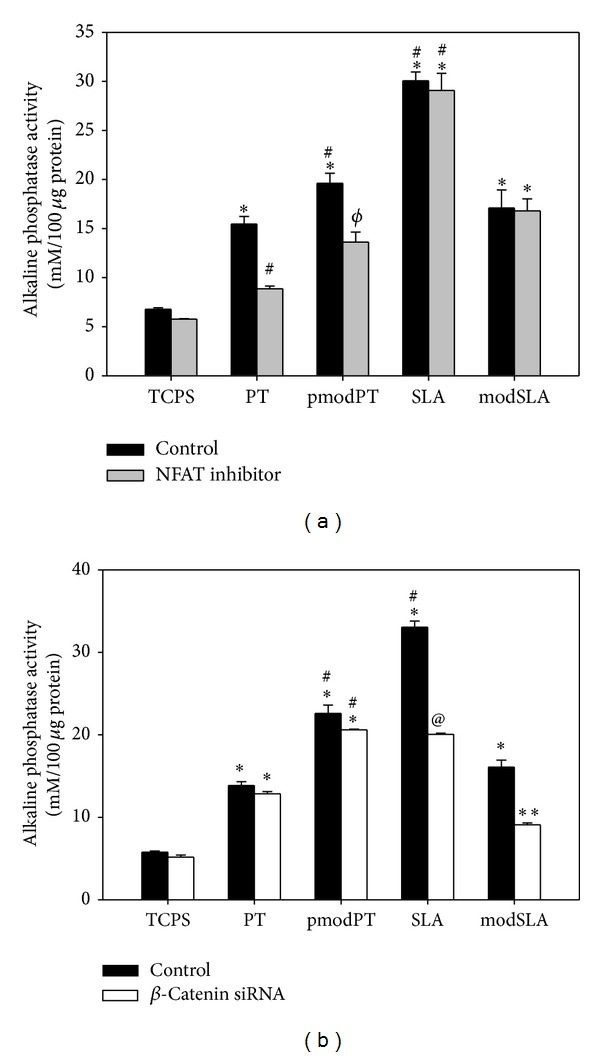
Effect of NFAT inhibitor or *β*-catenin knockdown on surface-dependent alkaline phosphatase (ALP) activity. (a) Cells were treated with the NFAT inhibitor VIVIT (500 nM) or (b) transfected with *β*-catenin-specific siRNA, and ALP activity was assessed after 4 days of periodontal ligament stem cell culture. A representative result from three independent experiments is shown. *P < 0.05 versus control (tissue culture polystyrene); ^#^ *P* < 0.05 versus pretreatment (PT) substrate; ^@^ *P* < 0.05 versus sand-blasted, large-grit acid-etched substrate; ^*ϕ*^ *P* < 0.05, versus modified PT substrate.

Subsequently, we assessed whether surface-specific activated canonical or noncanonical Wnt pathways influenced the osteogenic differentiation of PDLSCs. Nuclear factor of activated T cells (NFAT) inhibitor treatment decreased ALP activity of cells on PT and pmodPT surfaces but had no effect on SLA and modSLA surfaces, indicating that calcium-dependent Wnt signaling played a prominent role in regulating PDLSC osteogenesis on smooth surfaces (Figure 5(a)). However, when cells were transfected with *β*-catenin siRNA, ALP values were reduced significantly only on SLA and modSLA surfaces, suggesting the osteoinductive function of the canonical Wnt/*β*-catenin pathway on rough substrates (Figure 5(b)).

## 4. Discussion

In the present study, we provide experimental evidence that implant roughness and hydrophilicity can affect differential signaling molecules targeting the early stages of osteogenic differentiation of PDLSCs. Moreover, the PDLSC osteogenic response was regulated in a roughness-dependent manner, in which osteogenesis-related factors were increased on SLA surfaces compared with PT surfaces. However, increased hydrophilicity contributed to cell response in a different way; the osteogenic properties of PDLSCs were hydrophilicity dependent on PT, but not on SLA, surfaces. This unexpected response is not consistent with previous reports of increased osteogenic activity of cells on hydrophilic compared with conventional SLA surfaces* in vitro* and* in vivo *[9, 13]. The reason that the modSLA surface did not elicit the strongest PDLSC response remains unclear. Several recent studies have shown that not only roughness but also wettability can control osteoblast responses to a biomaterial [14, 15]. However, the precise roles of surface property are unclear and optimal implant characteristics are still debated. Moreover, many studies have used osteoblast and bone marrow-derived mesenchymal stem cell models with more differentiated osteogenic phenotypes than PDLSCs, indicating the cell-type specificity of responses on Ti substrates. This finding is also consistent with previous reports that matrix mineralization and proliferation were reduced significantly on textured surfaces compared with smooth surfaces in a murine femoral stromal cell system [16]. Moreover, immortalized PDL-hTERT cells show increased spreading, survival, and differentiation on smooth* versus* rough surfaces [10]. In a study conducted to develop a surface wettability gradient, the most water-wettable surfaces showed decreased osteoblast differentiation compared with less water-wettable surfaces [17]. Thus, these findings suggest very cell-type specific responses to different surface textures and hydrophilicity. We suggest that (1) our findings may be a consequence of reduced cell spreading, growth, and survival on the modSLA surface, indicating that hydrophilicity was not the only factor regulating biological cell responses; (2) PDLSCs recognize SLA surface conditions as an ideal environment for differentiation; and (3) the seemingly significant differences in responses to each surface between the* in vivo* and* in vitro* environments need further confirmation.

Cell fate depends on mutual extracellular signaling and the activation or repression of specific transcription factors that affect common intracellular signaling cascades. The gene expression analysis conducted in this study indicated that differential, substrate-dependent signaling activation may be responsible for the increased osteogenic activity of PDLSCs. We first identified the dependence of Wnt factor regulation on implant surfaces. The role of Wnt signaling in bone formation has been examined recently, and it is considered to be a fundamental signaling cascade for osteoblast differentiation [18]. Moreover, surface topography and chemistry have been shown to regulate Wnt signaling, a pivotal pathway for the commitment of mesenchymal stem cells to the osteoblast lineage [6]. Wnt signaling has several molecular pathways: the canonical Wnt pathway, which requires *β*-catenin, and the noncanonical Wnt pathways, which activates downstream signaling independent of *β*-catenin [19, 20]. Interestingly, our findings demonstrated that cell expression profiles of Wnt factors differed among Ti surfaces. Specifically, cells on rough SLA surfaces exhibited increased mRNA expression of the canonical Wnt signaling molecules Wnt3a and *β*-catenin, whereas smooth PT surfaces affected one noncanonical Wnt pathway, the calcium-dependent molecules Wnt5a, calmodulin, and NFATc1. Consistently, previous studies reported that different Wnt pathways were activated in response to individual implant properties [21–23]. Thus, implant topographical characteristics can modulate canonical and noncanonical pathways in various cell types, including PDLSCs.

In addition to Wnt signaling pathways, the present study classified several other molecules supporting the osteogenic response of PDLSCs to Ti surfaces. Among them, integrin *α*2 and *β*1 signaling is known to regulate the osteogenic factor osteoprotegerin and the integrin *α*2/*β*1 pair is required for osteoblast differentiation on microstructured Ti [24, 25]. Similarly, we found increased mRNA expression of integrin *α*2/*β*1 on SLA and modSLA substrates, consistent with the pattern of canonical Wnt signaling molecules, and decreased integrin expression with *β*-catenin siRNA, suggesting that the canonical pathways are involved in the regulation of integrins.

We have also demonstrated the different involvement of Shh/Gli and Notch signal transduction pathways with the different substrates. Each pathway has been suggested to play an important role in various cell types by regulating cell fate determination and differentiation [26–28]. Several studies have suggested that Shh/Gli and Notch signaling are important mechanisms involved in osteoblast differentiation and bone regeneration [27, 29, 30]. In dentistry-related research, cementogenesis by PDL cells on certain bioactive scaffolds was stimulated by activation of Wnt and Shh signaling pathways [31]. Moreover, a previous study evaluated surfaces with the immobilized Notch ligand Jagged-1; the osteogenic differentiation of human PDLSCs was increased significantly compared with untreated groups [32]. We also observed that blocking of canonical Wnt with *β*-catenin siRNA and noncanonical Wnt pathway with NFAT inhibitor decreased mRNA expression of Shh and Notch signaling molecules. Thus, our findings demonstrated that these signaling molecules involved in osteogenesis were differentially expressed according to implant properties and that Wnt signaling may act as an upstream regulator of the Shh and Notch pathways. Finally, ALP activity on smooth and rough substrates was inhibited by an NFAT inhibitor (blocking calcium-dependent Wnt5a) and a *β*-catenin knockdown using siRNA (blocking canonical Wnt/*β*-catenin), respectively. These results suggest that differentially activated Wnt pathways, depending on Ra and hydrophilicity, play important roles in the osteoinductive activity of PDLSCs.

These findings show that implant properties exert complex modulation of PDLSC differentiation through these various pathways. In future studies, the interactions of these pathways will be explored in detail. In conclusion, the present study showed that Ti implant surfaces can increase the osteogenic capacity of PDLSCs with no added osteoinductive factor and suggest what kinds of surface topography and chemistry may be optimal for PDLSCs. Moreover, we suggest that the many signaling molecules may play roles in surface-induced osteogenic differentiation of PDLSCs, and they may represent useful therapeutic targets for improving clinical performance and future cell-based implant engineering.

## Figures and Tables

**Table 1:** Primer sequences used for real-time RT-PCR analysis of gene expression.

Gene	Forward primer (5′-3′)	Reverse primer (5′-3′)
RUNX2	GTCTCACTGCCTCTCACTTG	CACACATCTCCTCCCTTCTG
OSX	TGAGGAGGAAGTTCACTATGG	TTCTTTGTGCCTGCTTTGC
FOSB	TCCAGGCGGAGACAGATCAGTTG	TCTTCGTAG GGGATCTTGCAGCC
FRA1	CCCTGCCGCCCTGTACCTTGTATC	AGACATTGGCTAGGGTGGCATCTGCA
Wnt3a	GTCCCGTCCCTCCCTTTC	ACCTCTCTTCCTACCTTTCCC
Wnt5a	TCTCAGCCCAAGCAACAAGG	GCCAGCATCACATCACAACAC
*β*-catenin	GGCAGCAACAGTCTTACC	TCCACATCCTCTTCCTCA
Integrin *α*2	ACTGTTCAAGGAGGAGAC	GGTCAAAGGCTTGTTTAGG
Integrin *β*1	ATTACTCAGATCCAACCAC	TCCTCCTCATTTCATTCATC
Calmodulin	CAGATATTGATGGAGACGGA	GAGCACACGAAGTACAAGAG
NFATc1	CCTTCGGAAGGGTGCCTTTT	AGGCGTGGGGCCTCAGCAGG
Shh	CGCCAGCGGAAGGTATGAAG	CAACTTGTCCTTACACCTCTGAGTC
Gli1	AATGCTGCCATGGATGCTAGA	GAGTATCAGTAGGTGGGAAGTCCATAT
Notch	GCCGCCTTTGTGCTTCTGTTC	CCGGTGGTCTGTCTGGTCGTC
Hes-1	AGGCGGACATTCTGGAAATG	CGGTACTTCCCCAGCACACTT
GAPDH	GCTCTCCAGAACATCATCC	TGCTTCACCACCTTCTTG

**Table 2:** Titanium surface roughness data.

	PT	SLA
Height parameters		
Sq (*μ*m)	0.1097	0.4151
Ssk	0.2341	−0.0547
Sku (*μ*m)	2.5841	2.4423
Sp (*μ*m)	0.3489	0.9488
Sa (*μ*m)	0.0889	0.3376
Hybrid parameters		
Sdq (rad)	0.28	1.8217
Sdr (%)	3.7307	85.24
